# *SOD1* Mutation Spectrum and Natural History of ALS Patients in a 15-Year Cohort in Southeastern China

**DOI:** 10.3389/fgene.2021.746060

**Published:** 2021-10-14

**Authors:** Lu-Xi Chen, Hai-Feng Xu, Pei-Shan Wang, Xin-Xia Yang, Zhi-Ying Wu, Hong-Fu Li

**Affiliations:** Department of Neurology and Research Center of Neurology in Second Affiliated Hospital and Key Laboratory of Medical Neurobiology of Zhejiang Province, Zhejiang University School of Medicine, Hangzhou, China

**Keywords:** amyotrophic lateral sclerosis, Cu/Zn superoxide dismutase 1, Chinese, Southeastern, mutation, natural history

## Abstract

**Background:** Mutations in superoxide dismutase 1 gene (*SOD1*) are the most frequent high penetrant genetic cause for amyotrophic lateral sclerosis (ALS) in the Chinese population. A detailed natural history of *SOD1*-mutated ALS patients will provide key information for ongoing genetic clinical trials.

**Methods:** We screened for *SOD1* mutations using whole exome sequencing (WES) in Chinese ALS cases from 2017 to 2021. Functional studies were then performed to confirm the pathogenicity of novel variants. In addition, we enrolled previously reported *SOD1* mutations in our centers from 2007 to 2017. The *SOD1* mutation spectrum, age at onset (AAO), diagnostic delay, and survival duration were analyzed.

**Results:** We found two novel *SOD1* variants (p.G17H and p.E134*) that exerted both gain-of-function and loss-of-function effects *in vitro*. Combined with our previous *SOD1*-mutated patients, 32 probands with 21 *SOD1* mutations were included with the four most frequently occurring mutations of p.V48A, p.H47R, p.C112Y, and p.G148D. *SOD1* mutations account for 58.9% of familial ALS (FALS) cases. The mean (SD) AAO was 46 ± 11.4 years with a significant difference between patients carrying mutations in exon 1 [*n* = 5, 34.6 (12.4) years] and exon 2 [*n* = 8, 51.4 (8.2) years] (*p* = 0.038). The mean of the diagnostic delay of FALS patients is significantly earlier than the sporadic ALS (SALS) patients [9.5 (4.8) vs. 20.3 (9.3) years, *p* = 0.0026]. In addition, male patients survived longer than female patients (40 vs. 16 months, *p* = 0.05).

**Conclusion:** Our results expanded the spectrum of *SOD1* mutations, highlighted the mutation distribution, and summarized the natural history of *SOD1*-mutated patients in southeastern China. Male patients were found to have better survival, and FALS patients received an earlier diagnosis. Our findings assist in providing a detailed clinical picture, which is important for ongoing genetic clinical trials.

## Introduction

Amyotrophic lateral sclerosis (ALS) is a progressive neurodegenerative disease characterized by the involvement of both upper and lower motor neurons in the spinal cord, brainstem, and motor cortex with or without cognitive dysfunction. ALS insidiously begins with focal weakness and muscle atrophy but relentlessly spreads to the diaphragm. Eventually the paralysis typically causes death in 3–5 years as a result of respiratory failure. Approximately 90% of ALS cases are sporadic (SALS), and the remaining cases are inherited (familial ALS or FALS) with a Mendelian pattern of inheritance, which implies that a single gene mutation can drive the pathogenesis of ALS.

The advancement of genetic technologies, such as whole exome sequencing (WES), has facilitated the identification of genes associated with ALS. Currently, more than 50 genes have been implicated in ALS. With joint efforts, genetic analysis has added valuable pieces to the ALS puzzle. Superoxide dismutase 1 gene (*SOD1*) was the first ALS-associated gene dating back to 1993 (Rosen et al., 1993) and led to engineering of the first transgenic model of *SOD1*-G93A mice (Gurney et al., 1994). It ushered in a new era of ALS research and set the stage for future genetic breakthroughs.

SOD1 is a ubiquitously expressed protein, existing as a homodimer of 32 kDa. Each monomer is highly structured, and intramolecular disulfide bridges increase their stability. However, mutations in *SOD1* can destabilize the protein and contribute to the collapse of the homodimeric structure and its subsequent aggregation (Brasil et al., 2019). It is well established that *SOD1* mutations lead to toxic gain of function of SOD1 proteins, but the effects of loss of function remain controversial (Gurney et al., 1994).

To date, more than 200 mutations in *SOD1* have been reported (http://www.hgmd.cf.ac.uk/). ALS patients carrying *SOD1* mutations present with a highly heterogenous phenotype that is clinically indistinguishable in SALS and FALS. *SOD1* gene mutations are the leading cause of FALS in the Chinese population. Therefore, it is important to clinically summarize both the mutation spectrum of the *SOD1* gene and the natural history of *SOD1*-mutated ALS patients in southeastern China.

## Materials and Methods

### Participants

Patients were recruited from the Second Affiliated Hospital of Zhejiang University School of Medicine from May 2017 to April 2021. A total of 114 patients with SALS and 15 with FALS were screened by WES. In addition, we performed the C9orf72 gene test, and all the hexanucleotide expansions of patients except one were within 30 repeats. For further phenotype–genotype analysis, we included previously reported *SOD1-*mutated probands in our centers from December 2007 to April 2017. All patients were diagnosed by at least two senior neurologists as having ALS according to the Revised El Escorial criteria (Brooks et al., 2000). All participants were of Han Chinese descent and came from southeastern China. The ethics committee of each participating center approved the study, and written consents were obtained from all participants during their first hospital visit. The clinical data of ALS patients, including gender, family history, age at onset (AAO), diagnostic delay, site of onset, side of onset, disease duration, and clinical manifestations were collected upon the first visit to the hospital.

### Whole Exome Sequencing, Bioinformatic Analysis, and Sanger Sequencing

Genomic DNA was extracted from blood using a DNA Extraction Kit (Qiagen, Hilden, Germany). The samples were captured by the Agilent Sure Select Human All Exon V6 products and sequenced on the Illumina HiSeq X Ten platform (XY Biotechnology Co. Ltd., Hangzhou, China). Further bioinformatic analysis has been performed according to our previously reported protocol (Dong et al., 2021). Briefly, all variants were annotated by ANNOVAR. SIFT and PolyPhen-2 software were used to predict the functional changes in proteins caused by the variants. Single Nucleotide Polymorphism (dbSNP) Database, the 1,000 Genomes Project, and the ExAC database were used to check the frequency in the general population. Variants were finally classified according to the American College of Medical Genetics and Genomics (ACMG) standards and guidelines. Sanger sequencing was performed to validate the potential variants. All primers covering the five exons in *SOD1* were seen in our previous report (Niu et al., 2011).

### Plasmid Construction

The coding sequence of human wild-type (WT) *SOD1* gene (RefSeq NM_000454.5) was cloned into the pFlag-CMV-4 vector using a ClonExpress II One Step Cloning Kit (Vazyme). Plasmids with mutant *SOD1* (c.400G > T and c.49_50del insCA) were created by PCR mutagenesis (Toyobo, Osaka, Japan) and were verified by Sanger sequencing. Four plasmids expressing Vector, WT, p.G17H, and p.E134* were constructed.

### Cell Culture and Transfection

HEK293T cells were maintained in Dulbecco’s modified essential medium DMEM-supplemented 10% fetal bovine serum stored in a humidified incubator under 5% CO_2_ at 37°C. Transient transfection was performed using Lipofectamine 3,000 (Invitrogen, Life Technologies, Grand Island, NY, United States ).

### Immunofluorescence Confocal Microscopy

HEK293T cells were seeded on poly-D-lysine (PDL)-treated coverslips (NEST, China) and transiently transfected with various expression plasmids (pFLAG-*SOD1*-WT, pFLAG-*SOD1*-G17H, pFLAG-*SOD1*-E134*). Twenty-4 hours later, the cells were rinsed with 1 × phosphate-buffered saline (PBS), fixed with 4% paraformaldehyde for 8 min, and then permeabilized with 1 × PBS supplemented with 0.01% Triton X100. The cells were blocked with 5% donkey serum and 1% bovine serum albumin (BSA; sigma, St. Louis, MO) in PBS for 1 h at room temperature. The primary antibody was rabbit antibody anti-flag (1:800, Cell Signaling Technology, 14793S). The secondary antibody was Alexa Fluor 488 donkey anti-rabbit. The cells were incubated with NucBlue Live Reagent (Hoechst 33342; Thermo Fisher Scientific, Oregon, United States) and visualized using Olympus FluoView FV3000 confocal microscopy with a ×63 objective.

### Western Blot

Forty-eight hours after transfection, HEK293T cells over-expressing pFlag-*SOD1*-WT, pFlag-*SOD1* mutants, or the empty vector were lysed and harvested. The protein samples were resolved by sodium dodecyl sulfate polyacrylamide gel electrophoresis (SDS-PAGE), transferred to polyvinylidene difluoride (PVDF) membrane, and blotted with the 5% non-fat milk. The antibodies against flag (1:1,000, CST) and β-tubulin (1:5,000) were used. All immunoblotting images were acquired using a BioRad system.

### Quantitative Real-Time PCR

Total RNA was extracted using Trizol reagent (Takara, Kusatsu, Japan), which was then reverse transcribed to cDNA by the PrimeScript RT reagent Kit (Takara, Kusatsu, Japan) in accordance with the manufacturer’s instructions. Reverse transcription (RT)-PCR was further performed using a SYBR Premix Ex Taq Kit (Takara, Kusatsu, Japan). The PCR conditions are listed as follows: incubation for 3 min at 50°C followed by incubation for 3 min at 95°C, and finally 40 cycles at a duration of 10 s each while at 95°C and then 30 s at 30°C. The following primers were used: target *SOD1* and human *GAPDH*. Detection and data analysis were conducted using an ABI StepOnePlus sequence detection system (Thermo Fisher Scientific, Oregon, United States ), and endogenous glyceraldehyde 3-phosphate dehydrogenase (GAPDH) was used as an internal control. Expression levels were quantified by threshold cycle values.

### Statistical Analysis

Descriptive statistics were provided for the site of onset, heredity of disease (FALS or SALS), gender, AAO, diagnostic delay, and survival time. Besides, continuous data were compared using Student’s t-test or the Mann–Whitney test, while dichotomous variables, such as gender and site of onset, were analyzed using either the standard chi-square test or Fisher’s exact test. Survival duration was determined by Kaplan-Meier analysis, and differences were determined by log-rank testing. A two-tailed *p* < 0.05 was considered statistically significant. All analyses were performed using GraphPad Prism 8.0 (GraphPad Software, CA, United States).

## Results

### Whole Exome Sequencing Screen of ALS Patients and Pathogenicity Classification of Novel *SOD1* Mutations

**FIGURE 1:**
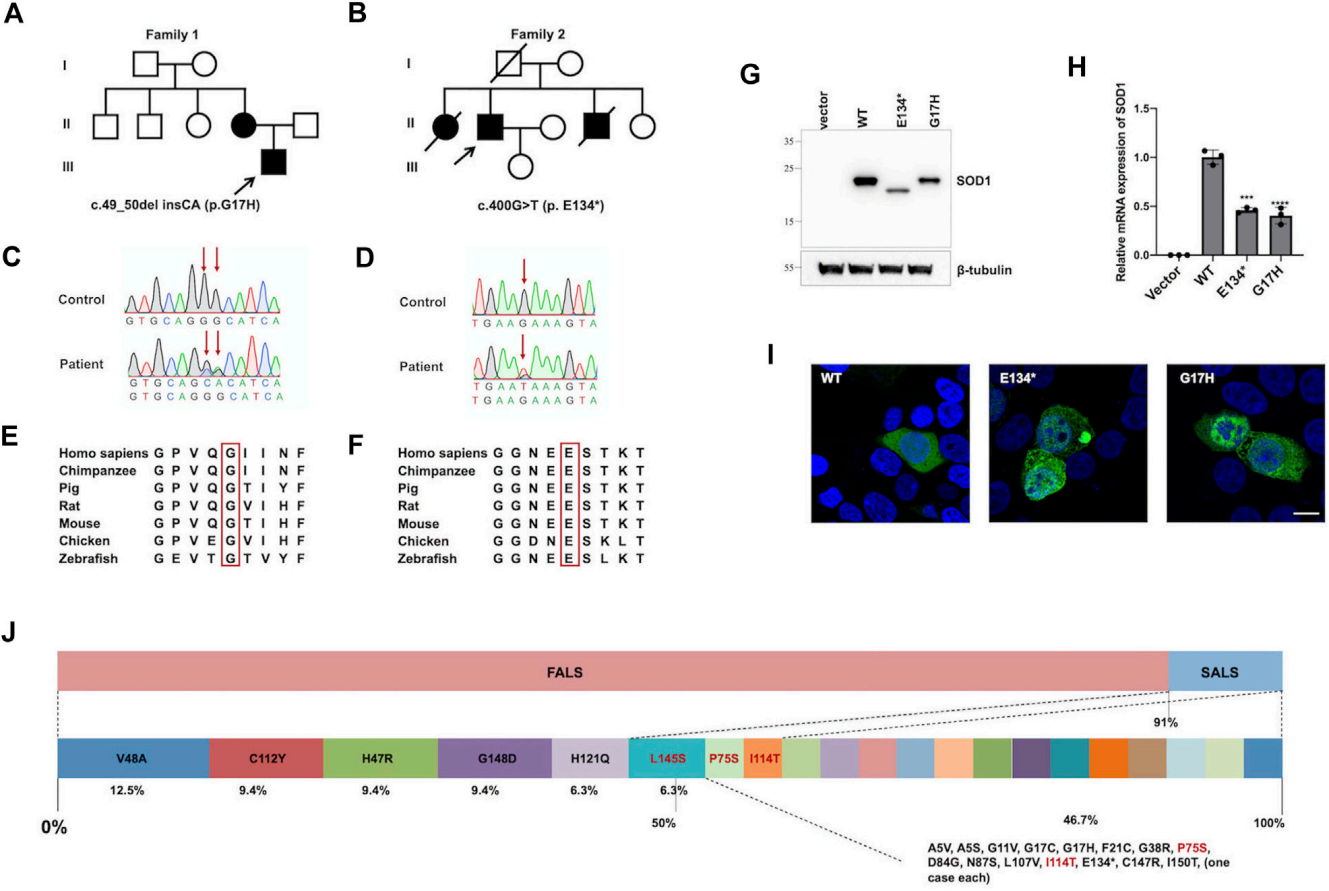
*SOD1* novel variants identified in patients, functional study, and *SOD1* mutation distribution. **(A,B)** Pedigrees of two ALS families carried missense variants of the *SOD1* gene; arrows indicate the proband of the family. **(C,D)** Sequence of c.49_50del insCA (p.G17H) and c.400G > T (p.E134*) variants in *SOD1* are shown; arrows indicate variant sites. **(E,F)** The p.G17H and p.E134* variants were highly conserved from chimpanzee to zebrafish. **(G)** HEK293T cells were transiently transfected with plasmids expressing Vector, p.WT, p.G17H, and p.E134*. The *SOD1* protein was detected by anti-Flag antibody. **(H)** Quantitative real-time PCR of *SOD1* mRNA levels in HEK293T cells transfected with empty vector, WT, or mutant flag-tagged *SOD1* vectors. GAPDH was used as an internal control. The data shown are representative of three independent experiments. Error bars indicate SDs, ***p < 0.001, ****p < 0.0001 (one-way ANOVA). **(I)** Immunofluorescence confocal of HEK293T cells expressing WT or mutant *SOD1*. **(J)** Mutation spectrum in *SOD1* gene of southeastern Chinese ALS patients in our ALS cohort.

From 2017 to 2021, a total of eight different variants in *SOD1* were found, including six known pathogenic variants (p.G38R, p.V48A, p.N87S, p.C112Y, p.I114T, and p.L145S) and two novel variants (p.G17H and p.E134*). Two heterozygous variants, c.49_50del insCA (p.G17H) and c.400G > T (p.E134*), were detected in two families with a positive family history (Figures 1A,B), which was confirmed with Sanger sequencing (Figures 1C,D). Both novel variants were absent in ESP6500 and ExAC database and were highly conserved from chimpanzee to zebrafish (Figures 1E,F). The nonsense variant, p.E134*, generates the truncated protein caused by premature termination. However, the nonsense variant was not regarded as very strong evidence of pathogenicity because loss of function is not the primary mechanism in *SOD1*-related ALS. In addition, p.E134* was predicted to be detrimental by Mutation Taster and CADD. The variants p.G17H and p.E134* were classified as likely pathogenic and pathogenic, respectively, according to ACMG criteria obtained using Varsome.

To further elucidate the biological effects of these two variants, SOD1 protein and mRNA levels of mutants were markedly reduced compared to WT (Figures 1G,H), indicating that the reduced protein expression was caused by reduced mRNA synthesis. The *in vitro* results showed that the mRNA levels of mutant *SOD1* were degraded more rapidly than WT, suggesting a loss-of-function effect. The aggregation propensity assay showed that the cells overexpressing WT plasmid showed diffuse cytoplasmic SOD1 protein, while the misfolded aggregates were seen in cells transfected with p.G17H and p.E134* mutants (Figure 1I). The soluble physiological SOD1 protein had a strong tendency to become toxic aggregates due to mutation, implying a gain-of-function effect.

### Mutation Frequency of *SOD1* in Our ALS Patients From Southeastern China

In this study, a total of 114 patients with SALS and 15 patients with FALS were screened by WES. Among them, 12 subjects were genetically identified as having *SOD1* variants, 10 (10/15, 66.7%) of whom were FALS patients and 2 (2/114, 1.8%) of whom were apparently SALS patients. We previously reported 20 probands with *SOD1* mutations in 36 unrelated FALS patients (Niu et al., 2011; Liu et al., 2014; Lin et al., 2019; Liu et al., 2019). When integrating these results into the current study, *SOD1* mutations account for 58.9% (32/51) of our FALS cases.

A total of 21 *SOD1* mutations, including 1 nonsense and 1 deletion/insertion mutations, were found spanning all five exons, but only one mutation was in exon 3 (Table 1). The most frequent *SOD1* mutation was p.V48A (4/32, 12.5%) in four FALS probands, followed by p.H47R, p.C112Y, and p.G148D [each were found in three probands (3/32, 9.4%)], as well as p.H121Q and p.L145S [each were found in two probands (2/32, 6.3%)], and the remaining mutations were each found in one proband (Figure 1J). Among all the mutations, p.L145S, p.P75S, and p.I114T are found in SALS patients (Figure 1J).

**TABLE 1:** The clinical features of the probands carrying mutations in *SOD1*.

Probands	Variant	Exon	Gender	Family history	AAO (y)	Diagnostic delay (m)	Site of onset	Side of onset	Disease duration (m)	Predominant features
1	A5S	1	M	F	29	3	LL	L	12	LMN dominance
2	A5V	1	F	F	47	8	UL	L	15	Classical ALS
3	G11V	1	F	F	25	9	LL	L	15	Classical ALS
4	G17C	1	M	F	49	8	spinal	L	38	LMN dominance
5	G17H	1	M	F	23	—	LL	R	36	Classical ALS
6	F21C	1	M	F	—	—	—	—	—	—
7	G38R	2	M	F	40	12	spinal	Both	>156A	Classical ALS
8	H47R	2	F	F	58	84	UL	R	180	LMN dominance
9	H47R	2	M	F	53	18	LL	Both	120	LMN dominance
10	H47R	2	F	F	55	18	LL	Both	>144	LMN dominance
11	V48A	2	F	F	53	20	LL	R	17	LMN dominance
12	V48A	2	F	F	42	12	UL	Both	>30A	LMN dominance
13	V48A	2	F	F	46	13	LL	R	>27A	Classical ALS
14	V48A	2	M	F	64	12	LL	Both	>25A	LMN dominance
15	P75S	3	M	S	59	23	LL	Both	65	LMN dominance
16	D84G	4	M	F	32		LL	R	70	LMN dominance
17	N87S	4	M	F	72	11	UL	R	>21A	Classical ALS
18	L107V	4	M	F	41	2	LL	R	10	Classical ALS
19	C112Y	4	M	F	47	9	UL	R	53	LMN dominance
20	C112Y	4	M	F	50	15	LL	R	40	LMN dominance
21	C112Y	4	M	F	47	6	LL	R	60	LMN dominance
22	I114T	4	M	S	60	10	UL	R	21	Classical ALS
23	H121Q	5	F	F	42	11	LL	Both	51	LMN dominance
24	H121Q	5	F	F	60	7	UL	L	39	Classical ALS
25	E134*	5	M	F	48	6	UL	R	>34A	Classical ALS
26	L145S	5	F	F	50	6	LL	Both	>31A	LMN dominance
27	L145S	5	F	S	48	28	LL	L	>46A	Classical ALS
28	C147R	5	F	F	39	3	bulbar	—	9	Classical ALS
29	G148D	5	F	F	34	4	UL	Both	12	LMN dominance
30	G148D	5	F	F	37	6	UL	L	15	Classical ALS
31	G148D	5	F	F	38	12	LL	L	14	Classical ALS
32	I150T	5	F	F	37	7	LL	Both	12	Classical ALS

Abbreviation: AAO, age at onset; UL, upper limb; LL, lower limb; LMN, lower motor neuron.

### Clinical Features and Natural History of ALS Patients Carrying the *SOD1* Mutation

Combining our previously reported *SOD1*-mutated patients in our centers, a total of 32 probands were genetically identified with *SOD1* mutations (Table 1). Twenty-nine (29/32, 90.6%) of the probands were FALS patients, and three (3/32, 9.4%) were apparently SALS patients. The gender ratio (M:F) was 1:1. With the exception of 1 case with missing data, only 1 patient among 31 available probands exhibited bulbar onset. The majority of them (18/31, 58%) presented with lower limb onset, but some (10/31, 33.3%) presented with upper limb onset. Sixteen probands presented with a predominantly lower motor neuron phenotype, while another 16 probands were the classical ALS phenotype.

**FIGURE 2:**
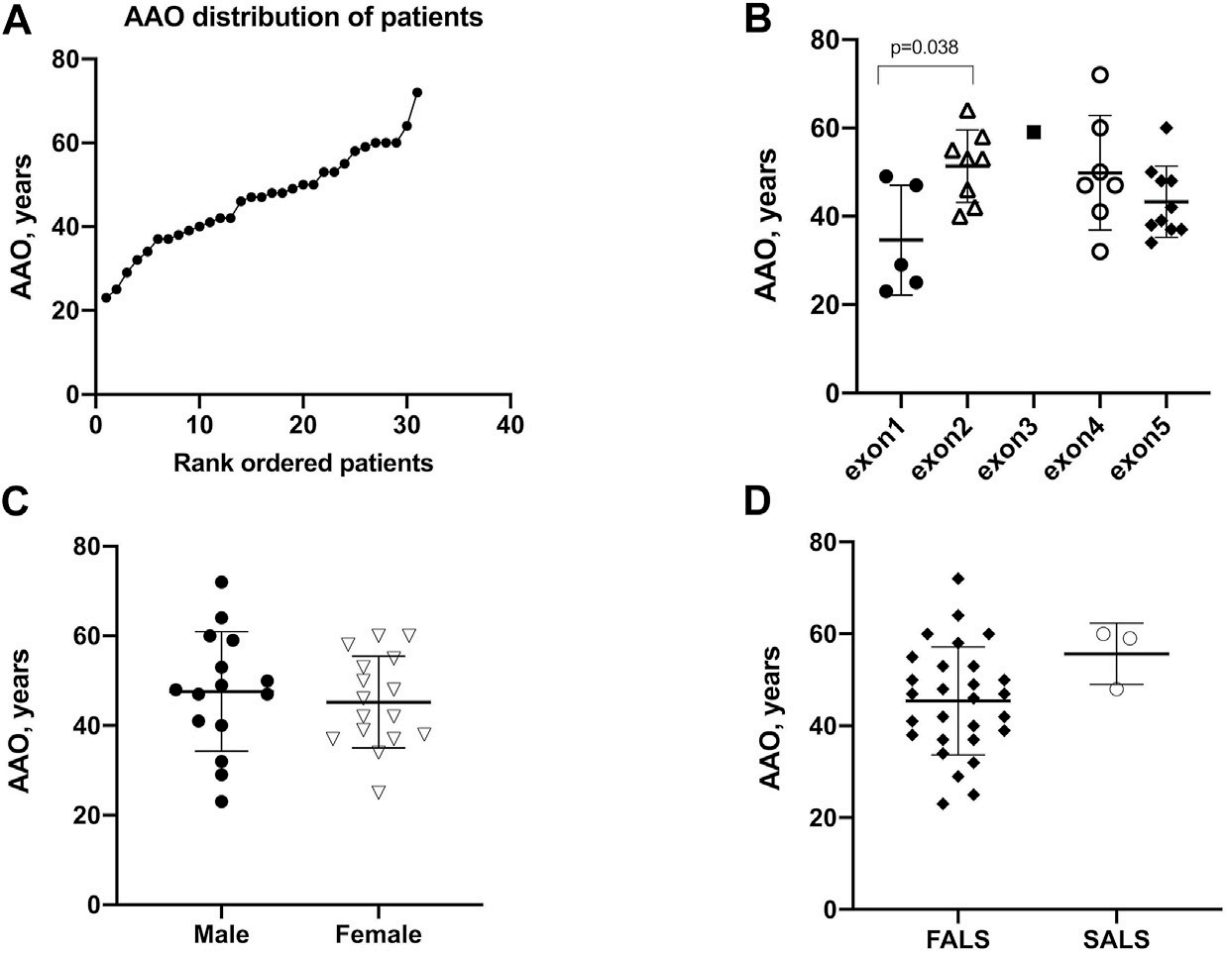
Age at onset (AAO) of patients with *SOD1* mutations. **(A)** Plot of rank ordered SOD1-mutant patients showing the median AAO of 47 years **(B)** Plot comparing the AAO among patients carrying mutations in different exons of the SOD1 gene. Patients (*n* = 24) harboring mutations in exon 1 were older than those (*n* = 18) harboring mutations in exon 2 (34.6 vs. 51.4 years, *p* = 0.038). **(C)** Plots comparing male and female patients and **(D)** plots comparing FALS patients with SALS patients; neither comparison identified a significant difference in AAO (*p* = 0.58 and 0.15, respectively).

With the exception of the patient with missing data, 31 patients had available AAO data for analysis. The mean (SD) AAO was 46 ± 11.4 years, and the median AAO was 47 years (Figure 2A). The mean AAO of patients carrying mutations in exon 1 [*n* = 5, 34.6 (12.4)] was statistically significantly earlier than those with mutations in exon 2 [*n* = 8, 51.4 (8.2)] (*p* = 0.038) (Figure 2B). There was no difference in the mean (SD) AAO between male and female patients [47.6 (12.2) vs. 45.25 (11.2), *p* = 0.58] (Figure 2C). The onset of FALS in patients is earlier than that of SALS, albeit without statistical significance [45.4 (11.4) vs. 55.7 (10.2), *p* = 0.15] (Figure 2D). The AAOs of ALS patients were highly heterogenous with the 25th and 75th percentiles at 38 and 53 years, respectively. Six mutations (p.A5S, p.G11V, p.G17H, p.D84G, p.G148D, and p.I150T) showed relatively younger AAOs, which was lower than the first quartile (25%, <38 years), and another six mutations (p.H47R, p.V48A, p.P75S, p.N87S, p.I114T, and p.H121Q) presented with relatively older AAOs, which were higher than the third quartile (75%, >53 years). The youngest and oldest AAOs are p.G17H and p.N87S, which were found in patients in their twenties and seventies, respectively.

**FIGURE 3:**
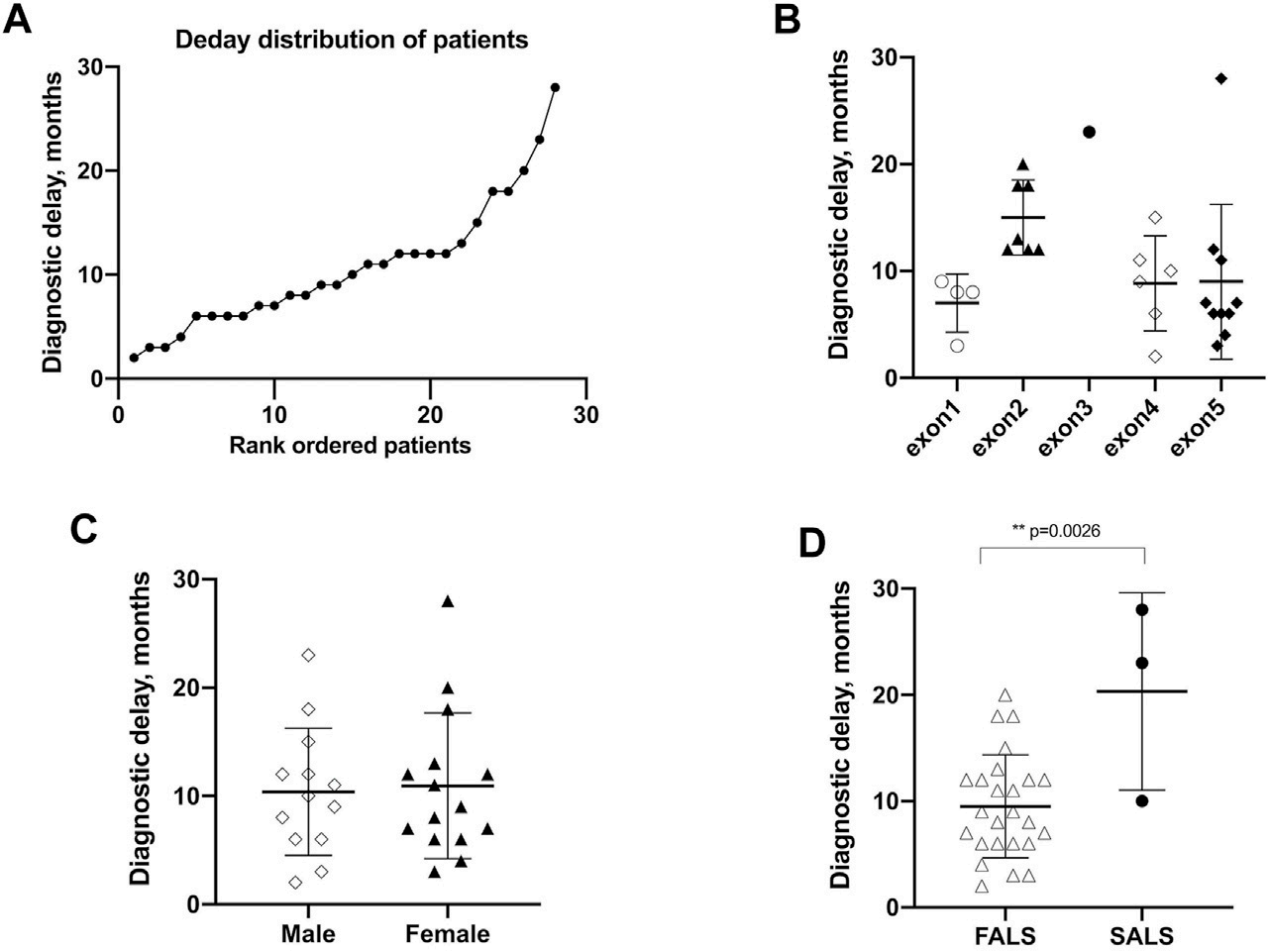
Diagnostic delay of patients with *SOD1* mutations. **(A)** Plot of rank ordered SOD1-mutant patients showing the median diagnostic delay of 9.5 months **(B)** Plot comparing the diagnostic delay showed no differences in different exons of *SOD1* gene. **(C)** Plots comparing the diagnostic delay exhibited no differences between male and female patients. **(D)** plots comparing FALS patients with SALS patients in the diagnostic delay time; neither comparison identified a significant difference in AAO (*p* = 0.58 and 0.15, respectively). FALS patients (*n* = 25) received an earlier diagnosis than SALS patients (*n* = 3) [9.5 (4.8) vs. 20.3 (9.3), *p* = 0.0026].

Twenty-nine probands patients were accessible for data regarding diagnostic delay. With the exception of one case with an extremely long diagnostic delay of 84 months, the mean (SD) diagnostic delay of the remaining was 10.7 ± 6.2 months (Figure 3A). The diagnostic delay showed no differences in different exons (Figure 3B). The time to diagnosis of male patients exhibited no difference compared to female patients [10.4 (5.9) vs. 11.0 (6.7) months, *p* = 0.82] (Figure 3C). It is worth noting that the mean of the diagnostic delay of FALS patients is less than that of SALS patients and was found to be statistically significant [9.5 (4.8) vs. 20.3 (9.3) months, *p* = 0.0026] (Figure 3D). Diagnostic delay varied greatly across different mutations. Four mutations of four probands, namely, p.A5S, p.L107V, p.C147R, and p.G148D, showed a diagnostic delay less than the first quartile (25%, <6 months). Another five mutations of eight probands, namely, p.H47R, p.V48A, p.P75S, p.C112Y, and p.L145S, showed a diagnostic delay more than the third quartile (75%, >12 months).

**FIGURE 4:**
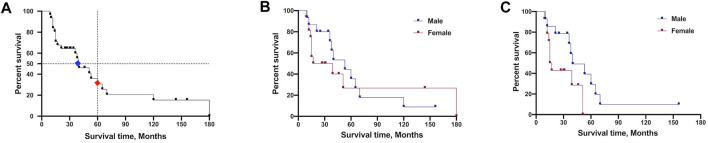
Survival analysis comparison of patients carrying *SOD1* mutations. **(A)** Plot of survival probabilities for all patients with *SOD1* mutations. The overall median survival time is seen in blue mark (40 months), and the 5-years survival rate is seen in red (30.7%). **(B)** Plot of survival probabilities between all male and female patients (median survival 53 vs. 28 months, *p* = 0.63). **(C)** Plot of survival probabilities between male and female patients after excluding the H47R mutation data, indicating that males had a longer survival time than females (median survival 40 vs. 16 months, *p* = 0.05).

Disease duration data were available for 31 probands, and 9 patients still survive at the censoring date. The median survival time was 40.0 months, and the 5-years survival rate was 30.7% for all subjects (Figure 4A). The survival time showed no difference between all male and female patients (Figure 4B). Given the extremely long survival time in mutation of p.H47R, we excluded the p.H47R mutation in both male and female patients for statistical analysis. Strikingly, male patients showed significantly longer survival time than female patients (40 vs. 16 months, *p* = 0.05) (Figure 4C).

## Discussion

In this study, which was combined with our previously reported *SOD1* mutations, 32 ALS probands were harboring 21 confirmed *SOD1* mutations. This is the first study to study *SOD1* mutations to date in the southeastern Chinese ALS population. The extensive analysis of *SOD1* mutation distribution and natural history could serve ongoing clinical trials targeted for patients with *SOD1* mutations.

Both novel variants (p.G17H and p.E134*) could form toxic aggregates *in vitro*, indicating that they caused loss of protein intrinsic stability. Interestingly, a loss-of-function effect was also shown in both variants, given the reduced protein expression and mRNA synthesis. Many *SOD1* mutations, such as p.G93A, p.G37R, and p.H48Q, would increase protein expression, which is in accordance with the gain-of-function presumption (Tang et al., 2019). However, overloading misfolded SOD1 proteins would induce endoplasmic reticulum (ER) stress, subsequently activating the unfold protein response (UPR) to maintain homeostasis. Once the UPR is activated, overall protein translation would shut down to alleviate ER stress by stopping the translation of misfolded proteins. Our results highlight that albeit with reduced expression of toxic protein level, neurons still fail to control the homeostasis because of toxic SOD1 aggregated protein.

In southeastern Chinese *SOD1* mutant patients with ALS, the four most frequent mutations in *SOD1* gene were p.V48A (4/32, 12.5%), H47R, C112Y, and G148D (3/32, 9.4%). The p.V48A has been reported by another team from southern China (Chen et al., 2020), implying that there might be a founder effect existing for p.V48A in southeastern China. According to a recent nationwide survey on *SOD1* in China by Fan’s team, p.H47R was the most frequent mutation in China (Tang et al., 2019) and Japan (Yamashita and Ando, 2015). In our study, patients with p.H47R and p.C112Y variants presented with the typical *SOD1*-related phenotype (lower limbs onset and lower motor neuron involvement) and mild disease course (longer diagnostic delay and extended survival time) (Table 1). The most predominant *SOD1* gene mutations in total were p.D91A, followed by p.I114T and p.A5V (Yamashita and Ando, 2015). The mutation p.D91A was absent in our study and absent in southeastern Chinese demographic, and only one case with p.A5V mutation was found. However, approximately 30% of all Finnish ALS patients have p.D91A mutation, and the p.A5V accounts for almost half in *SOD1* mutations in the United States (Bali et al., 2017; Korpi et al., 2020). Notably, SOD1^A5V^ is a relatively homogeneous form exclusively with aggressive progression and short disease duration (Bali et al., 2017). Therefore, the frequency of specific mutations can vary among different regions.

ALS predominance in males was reported by several teams in Mainland and Taiwan in China (Soong et al., 2014; Wei et al., 2017; Tang et al., 2019), as well as the largest cohort study from North America (Bali et al., 2017). However, there was no gender difference in our patients harboring *SOD1* mutations probably due to the limited sample size. The median survival time of *SOD1*-mutated patients in our study is 3.3 years, while it is 2.7 years in American patients (Bali et al., 2017). The aggressive mutation p.A5V is an unignorable bias for survival time calculation which accounts for 38% of participants in the cohort of North America (Bali et al., 2017). And the benign form p.H47R was the most frequently identified mutation in *SOD1*-mutated Chinese patients. Therefore, it is reasonable to find different survival times in Chinese and American patients. The 5-years survival rate was 30.7% for all subjects in our study compared to 55% in Fan’s team (Tang et al., 2019). In addition, our results revealed that male *SOD1*-mutated patients had longer survival time, which is not consistent with the finding by Fan’s team (Tang et al., 2019). At first, we found that the survival time showed no difference between genders when including all variants (Figure 4B). Because the variant p.H47R is associated with long survival time and high frequency in Chinese *SOD1*-mutated patients, we think it would be a potential bias to affect the statistical analysis. Therefore, we further analyzed the survival time by excluding the p.H47R variant in one male and two female patients, and the results revealed increased survival in male patients (Figure 4C). Given the variant p.H47R predominance in females in the study conducted by Fan’s team (Tang et al., 2019), this may be the possible reason for this conflicting result. Thus, it deserves our attention when combining p.H47R into the overall results, especially when considering the limited sample size. One possible explanation for gender differences might be the effect of gonadal hormones. A very recent report demonstrated that dihydrotestosterone (DHT) levels were significantly decreased in all ALS patients, indicating that DHT is probably integral to motor neuron survival (Sawal et al., 2020).

The mean (SD) AAO of the *SOD1*-mutant patients in this study was 46 ± 11.4 years, which is older than the finding from the largest study of a Chinese population (43.92 ± 9.24) but younger than the multi-center cohort from North America (49.7 ± 12.3) (Bali et al., 2017; Tang et al., 2019). Moreover, the mean AAO of the SOD1-mutant patients is younger than that of the overall Chinese ALS population (55.5–58.76 years) (Liu et al., 2018). *SOD1*-mutant patients were a subgroup in the overall ALS population, and similar findings could be seen in Japan (62.1 years) (Furuta et al., 2013), Europe (62.1–66.3 years) (Al-Chalabi et al., 2014), and Canada (48.9 years) (Eisen et al., 2008). Interestingly, the overall AAO in India is 46.2 years old, which is lower than many regions in the world (Nalini et al., 2008). The mean (SD) diagnostic delay of *SOD1*-mutant patients was 10.7 ± 6.2 months, which is shorter than 14 months of overall Chinese ALS patients (Chen et al., 2021). The *SOD1*-mutant FALS patients received an earlier diagnosis than the SALS patients (Figure 3D), which is not consistent with the finding of Fan’s team. All ALS patients in our cohort carried one mutation in the *SOD1* gene. However, it should be mentioned that 3.8–4.7% of ALS patients carried potentially pathogenic variants in more than one ALS genes (Cady et al., 2015; Liu et al., 2016). Most FALS patients carried missense mutations in *SOD1* concomitantly with the variants in other genes, including *SETX*, *OPTN*, *DAO*, *GRN*, *ANG*, and *DCTN1* (Cady et al., 2015; Liu et al., 2016). The average AAO was less than 10 years earlier in patients harboring multiple variants compared with those harboring one (Cady et al., 2015; Liu et al., 2016). However, the burden of variants in ALS-related genes seemingly did not influence the site of onset or survival (Cady et al., 2015; Liu et al., 2016).

### Limitations

The *SOD1*-mutant ALS patients in this cohort all come from southeastern China; thus, it should be mentioned that these data are not representative of all Chinese populations. Given the limited sample size, the frequency of *SOD1* for FALS probably was overestimated. Moreover, our center is still working on the enrollment of the ALS patients and screening for the genetic causes. In addition, for those patients with family history but negative genetic results, we would need a larger scale sequence such as whole genome sequencing (WGS) to define the genetic information of Chinese ALS cases.

## Conclusion

Overall, our work provides an updated natural history of *SOD1*-mutated ALS patients in southeastern China, which can serve as a supplementary reference database for *SOD1*-targeted therapy in clinical trials.

## Data Availability

The original contributions presented in the study are included in the article/Supplementary Material, further inquiries can be directed to the corresponding authors. The data presented in the study are deposited in the Sequence Read Archive repository, accession number SAMN21561907.
